# DYRK1A activates NFATC1 to increase glioblastoma migration

**DOI:** 10.1002/cam4.4159

**Published:** 2021-07-26

**Authors:** Heng Liu, Qian Sun, Shuai Chen, Long Chen, Wenming Jia, Juan Zhao, Xiulian Sun

**Affiliations:** ^1^ NHC Key Laboratory of Otorhinolaryngology Qilu Hospital of Shandong University Jinan Shandong China; ^2^ Department of Otorhinolaryngology Qilu Hospital of Shandong University Jinan Shandong China; ^3^ Immunology Institute School of Basic Medical Sciences Cheeloo College of Medicine Shandong University Jinan Shandong China; ^4^ Brain Research Institute Qilu Hospital of Shandong University Jinan Shandong China

**Keywords:** DYRK1A, glioblastoma, inhibitor, migration, NFATc1

## Abstract

Glioblastoma (GBM) is the most aggressive glioma, and is prone to develop resistance to chemotherapy and radiotherapy; hence, patients with glioblastoma have a high recurrence rate and a low 1‐year survival rate. In addition, the pathogenesis of glioblastoma is complex and largely unknown, and the available treatments are limited. Here, we uncovered a fundamental role of DYRK1A in regulating NFATC1 in GBMs. We found that DYRK1A was highly expressed in glioma and glioblastoma cells, and its expression was positively correlated with that of NFATC1. Moreover, inhibition of DYRK1A promoted NFATC1 degradation in GBM cells and sharply reduced the transactivation of NFATC1, not only by decreasing the expression of NFATC1‐targeted genes, but also by reducing the luciferase activity, and vice versa. However, DYRK1A had the opposite effect on NFATC2. Most importantly, our data suggest that DYRK1A inhibition reduces glioblastoma migration. Polypeptides derived from the DYRK1A‐targeted motif of NFATC1, by competitively blocking DYRK1A kinase activity on NFATC1, clearly destabilized NFATC1 protein and impaired glioblastoma migration. We propose that the recovery of NFATC1 stability is a key oncogenic event in a large proportion of gliomas, and pharmacological inhibition of DYRK1A by polypeptides could represent a promising therapeutic intervention for GBM.

## Graphical abstract

We found that DYRK1A protein was positively correlated with that of NFATc1 through affecting NFATc1 degradation and transactivation in GBM cells. DYRK1A inhibition or polypeptide derived from DYRK1A‐targeted motif of NFATc1, clearly destabilized NFATc1 protein and impaired glioblastoma migration. We propose that the recovery of NFATc1 stability is a key oncogenic event in a large proportion of gliomas and polypeptide pharmacological inhibition of DYRK1A could represent a promising therapeutic intervention for NFATc1‐dependent GBM.

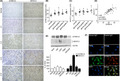

## 1 INTRODUCTION

Glioma is the most common brain cancer, and high‐grade gliomas, including glioblastoma multiforme (GBM), are the most aggressive types of glioma.^1^ The current standard treatment for GBM involves surgical resection followed by the administration of an alkylating agent, temozolomide, both concurrent with and after radiotherapy. Bevacizumab is administered as a second‐line treatment after relapse.^2^, ^3^ However, GBM is resistant to chemotherapy and radiotherapy, with a median patient survival time of approximately one year.^1^, ^4^ The highly invasive nature of GBM cells is thought to contribute to the poor prognosis of this tumor, necessitating the exploration of alternative therapies. Many factors are involved in the migration and invasion of malignant tumors, including the nuclear factor of activated T cells (NFAT).^5^, ^6^, ^7^

NFAT, originally found in activated T cells, is a transcription factor that induces gene transcription during the immune response.^8^ The NFAT transcription factor family consists of five members: NFAT1 (NFATC2, NFATp), NFAT2 (NFATC1, NFATc), NFAT3 (NFATC4), NFAT4 (NFATC3, NFATx), and NFAT5 (TonEBP, OREBP). NFAT is widely expressed in mammalian tissue cells essential for development, differentiation, and immune response.^9^, ^10^, ^11^, ^12^, ^13^, ^14^, ^15^ As basic factors in multiple‐signal transduction pathways in cells, the NFAT family may regulate the expression levels of multiple cytokines, such as interleukin 2 (IL2), interleukin 4 (IL4), interferon gamma (IFNG), tumor necrosis factor alpha (TNFα), and prostaglandin‐endoperoxide synthase 2 (PTGS2).^16^ Recent studies have shown that NFAT also has carcinogenic potential.^2^, ^17^, ^18^ In particular, NFATC1 may act as an oncogenic factor, whereas NFATC2 acts as a tumor suppressor.^19^, ^20^ However, little attention has been given to the transcriptional activity of NFATC1 involved in GBM.^21^

Different protein kinases are involved in the regulation of NFAT transcriptional activity, including the mitogen‐activated protein kinase family (MAPKs, such as MAPK14, MAPK8, and MAPK1), glycogen synthase kinase 3 beta (GSK3B), protein kinase A (PKA), casein kinase 1 alpha 1 (CSNK1A1), mitogen‐activated protein kinase kinase 1 (MAP2K1), and the dual‐specificity tyrosine‐phosphorylation‐regulated kinase (DYRK) family, including DYRK1A and DYRK2. In most cases, the phosphorylation of NFAT causes its export from the nucleus and loss of transcriptional activity. However, there is also evidence that NFAT phosphorylation increases its transcriptional activity. For example, MAPK8 and MAP3K8 activate NFATC2, while MAPK9, MAPK14, and PIM1 activate NFATC1. In contrast, MAPK8 inhibits NFATC1, whereas MAPK9 and MAPK14 inhibit NFATC2.^22^, ^23^, ^24^, ^25^, ^26^, ^27^, ^28^, ^29^ In our previous study, we demonstrated that DYRK1A phosphorylated NFATC1/A at S261, S278, S403, and S409 and interfered with NFATC1 ubiquitination and ubiquitin‐proteasome degradation, resulting in increased NFATC1 protein stability, in contrast to decreased NFATC2 protein stability caused by DYRK1A.^30^

In this study, we demonstrate that glioma tissues and glioblastoma cells with high expression levels of DYRK1A exhibit strong NFATC1 activity, revealing a remarkably positive correlation between DYRK1A and NFATC1. DYRK1A increases NFATC1 transcriptional activity; however, its effect is not the same on NFATC2. Inhibition of DYRK1A also reduces GBM migration. Finally, we demonstrate that blocking peptides competitively inhibit the effect of DYRK1A on NFATC1, clearly reducing NFATC1 protein and impairing tumor migration. Our results suggest that DYRK1A is a potential therapeutic target for glioma migration.

## 2 MATERIALS AND METHODS

### 2.1 Cell culture and reagents

NHA, U87, U251, T98G, and HEK293 cells were cultured at 37°C in an incubator containing 5% CO_2_, as previously described.^30^ Cell transfection was conducted using Lipofectamine 2000 (11668–027, Thermo Fisher Scientific, Inc.) according to the manufacturer's guidelines. Peptides were synthesized by China Peptides Corporation.

### 2.2 Plasmid construction

Plasmids of WT NFATC2, NFAT/AP‐13 × luciferase, and pRL‐TK were obtained from Addgene (Cambridge). The p‐NFATC1mycflag, pCMV‐DYRK1A, and pGFP‐V‐RS‐shDYRK1A were constructed in our laboratory.^30^

### 2.3 Dual luciferase assay

Luciferase activity was determined following a protocol supplied with the Dual‐Luciferase^®^ Reporter Assay System (E1910, Promega Biotech Co.), as described previously.^31^

### 2.4 Quantitative RT‐PCR

Total RNA was extracted from the cells using TRIzol^®^ Reagent (15596026, Thermo Fisher Scientific, Inc.) 48 h after transfection. An RT‐PCR kit (K1005S, Promega Biotech Co.) was used to synthesize the first strand of cDNA from equal amounts of total RNA samples, and real‐time fluorescence PCR was performed with SYBR Green Realtime PCR Master Mix (QPK‐201, TOYOBO Co., Ltd., Kita‐ku, Osaka, Japan) according to the manufacturer's protocol. Primers of TNFα F (5′‐GAGGCCAAGCCCTGGTATG‐3′) and TNFα R (5′‐CGGGCCGATTGATCTCAGC‐3′) were used to amplify a 91‐bp fragment of the TNFα gene. Primers of IL2 F (5′‐AGAACCCGAAACTGACTCGT‐3′) and IL2 R (5′‐AGGCATTGCAGGTGTTTCAG‐3′) were used to amplify an 82 bp fragment. A 96‐bp fragment of human ACTB amplified with primers 5′‐CCCTGGAGAAGAGCTACGAG‐3′ and 5′‐ GGAAGGAAGGCTGGAAGAGT‐3′ was used as an internal control. The fold‐change in gene expression was calculated by the 2‐ΔΔCT method following normalization to ACTIN.

### 2.5 Western blot

Western blotting was performed as previously described.^31^ Monoclonal anti‐flag M2 antibody (F1804; Merck KGaA) was used to detect exogenous NFATC1. NFATC1 monoclonal antibody (MA3‐024, Thermo Fisher Scientific, Inc.) and NFATC2 monoclonal antibody (MA1‐025, Thermo Fisher Scientific, Inc.) were used to detect NFATC1 and NFATC2, respectively. DYRK1A polyclonal antibody (2771, Cell Signaling Technology, Inc.) was used to detect DYRK1A, and an anti‐β‐actin monoclonal antibody (A1978, Merck KGaA) was used to detect ACTB as a loading control. IRDye 680 goat anti‐rabbit IgG (c10207‐01, Li‐COR, Lincoln) and IRDye 800CW goat anti‐mouse IgG (C11026‐03, Li‐COR, Lincoln) were used as secondary antibodies. Detection and quantification were performed using the Li‐COR Odyssey imaging system.

### 2.6 Cell migration assay

Millicell hanging cell culture inserts (PIEP15R48, Millipore) were placed in a 12‐well cell culture receiver plate (PIMWS1250, Merck KGaA). The cells were trypsinized 24 h after transfection, washed twice, and counted to 4 × 10^5^/mL with an FBS‐free medium. Five hundred microliters of cell suspension was added to the top chamber, and 1 mL medium with 20% FBS was added to the lower chamber of the plate. Cells on the top side of the filter were removed by scrubbing twice with cotton‐tipped swabs moistened with FBS‐free medium. Cells on the underside of the filter were mounted in VECTASHIELD mounting medium with DAPI (H1200, VECTOR Labs), and were counted using a Leica DMI4000 B microscope.

### 2.7 Tissue microarray

The tissue microarrays (GL481 and T173a) were stained using techniques and reagents from US Biomax. In brief, the slides were deparaffinized, and antigens were retrieved using microwave antigen retrieval. Endogenous oxidase was removed using H_2_O_2_ and blocked with serum. The slides were stained with primary antibodies, including monoclonal antibodies against NFATC1 (MA3‐024, Thermo Fisher Scientific, Inc.) and polyclonal rabbit antibody against DYRK1A (2771, Cell Signaling Technology, Inc.). The slides were then incubated with mouse‐ or rabbit‐specific secondary antibodies labeled with biotin and streptavidin and covalently linked in turn to horseradish peroxidase. Finally, the slides were developed with diaminobenzidine. Immunohistochemistry images were captured using a Leica DM2500 fluorescent microscope and analyzed with ImageJ software as follows: the original images were inverted and the background was subtracted. The mean intensities were measured to reflect the immunohistochemical expression signals.

### 2.8 Immunostaining

U251 cells were seeded in a glass‐bottom dish. When cells were 30%–50% confluent, immunostaining was performed as previously described.^31^ The cells were fixed in 4% paraformaldehyde and permeabilized with 0.1% PBST. Then, the cells were blocked with 5% BSA and successively incubated with primary and secondary antibodies in 5% BSA–0.1% PBST. Finally, the stained cells were mounted in VECTASHIELD mounting medium with DAPI (H1200, VECTOR Labs), and the images were captured with LIONHEART FX (BioTek). NFATC1 monoclonal antibody (MA3‐024, Thermo Fisher Scientific, Inc.) was used to detect NFATC1. DYRK1A polyclonal antibody (2771, Cell Signaling Technology, Inc.) was used to detect DYRK1A. Mouse IgG (Santa Cruz, sc‐2025) and rabbit IgG (Proteintech, B900610) were used as negative controls. CoraLite488—conjugated Affinipure goat anti‐mouse IgG (H+L) (SA00013‐1, Proteintech, Wuhan, Hubei, P.R.C) and CoraLite594—conjugated goat anti‐rabbit IgG (H+L) (SA00013‐4, Proteintech) were used as secondary antibodies.

### 2.9 Cytokine antibody array

Cytokine antibody analysis was conducted using a human cytokine antibody array (AAH‐CYT‐7–4, RayBiotech) according to the manufacturer's protocol. Briefly, the membrane was blocked with a blocking buffer for 30 min. After washing, the cell culture supernatant was added to the membrane and incubated for 2 h at RT, followed by incubation with biotin‐conjugated antibodies and IRDye 800CW Streptavidin (926–32230, Li‐COR). The labeled proteins were observed and quantified using the Li‐COR Odyssey imaging system.

### 2.10 Data analysis

All experiments were repeated three times. Representative blots are shown in the figures for western blotting (WB). Gray values of the images were analyzed using ImageJ 1.46r software. Values are presented as mean ± SE. An independent‐samples t‐test was performed for statistical analysis, and Wilcoxon signed‐rank tests were used if the data did not follow a Gaussian distribution. For multigroup comparisons, one‐way analysis of variance (ANOVA) followed by Bonferroni's post hoc tests were performed. All experimental data were analyzed using GraphPad Prism 5 software (GraphPad). All statistical tests were two‐sided, and *p*‐values less than 0.05 were defined as statistically significant.

## 3 RESULTS

### 3.1 DYRK1A and NFATC1 are highly and coordinately expressed in glioma

**FIGURE 1:**
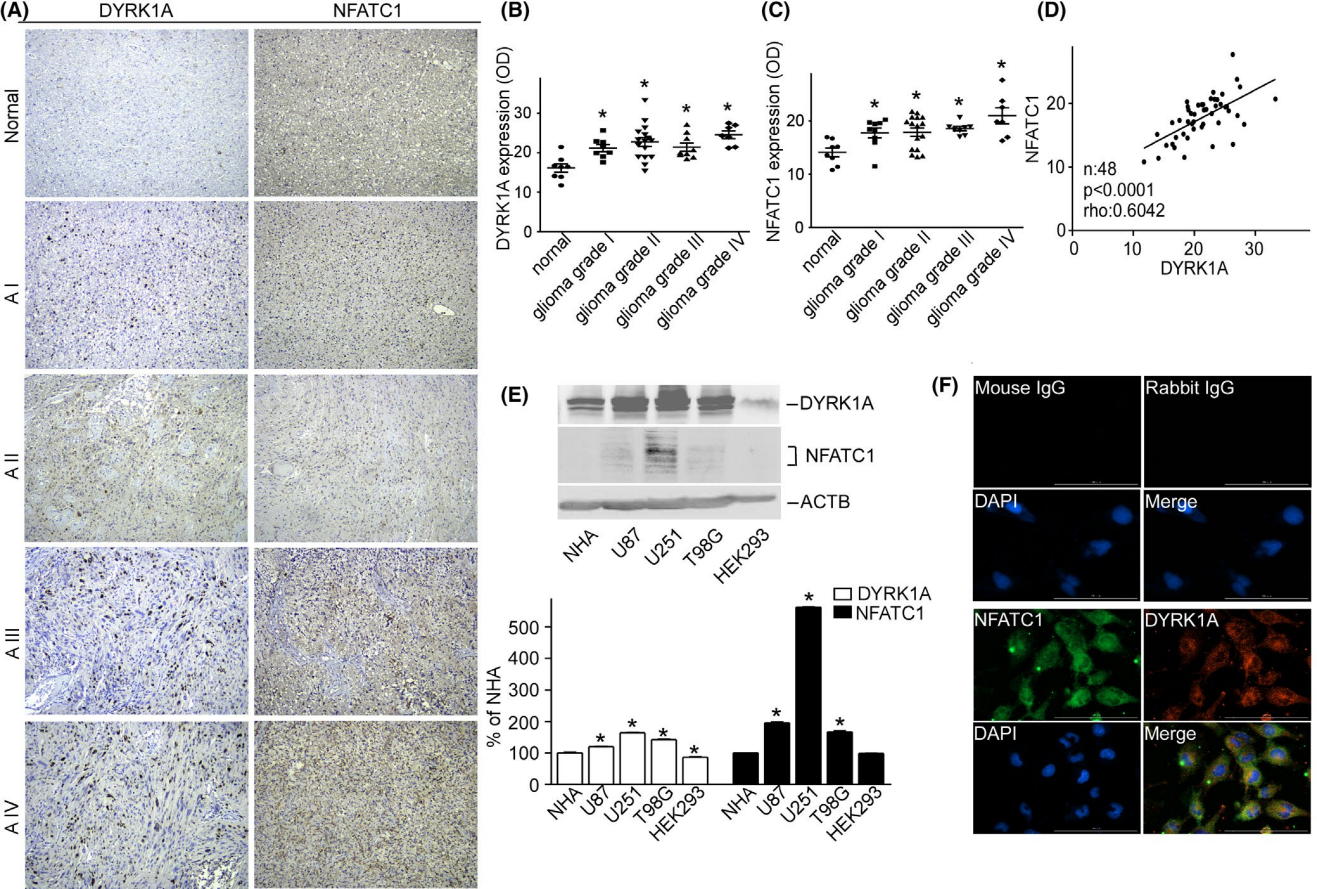
DYRK1A and NFATC1 protein expression were coordinately increased in brain gliomas. A Representative images for the immunohistochemical staining of DYRK1A and NFATC1 in four different grades of gliomas and normal tissues. DYRK1A was detected using a polyclonal anti‐DYRK1A antibody (CST, #2771) and NFATC1 was detected using a monoclonal antibody (Thermo Fisher, #MA3‐024) in the microarray (Biomax, #GL481 and T173a). B. DYRK1A protein expression was increased in gliomas. The expression of DYRK1A was quantified by ImageJ software as described in the methods section. C. NFATC1 protein levels were increased in gliomas. The expression of NFATC1 was quantified by ImageJ software as described in the methods section. D. Correlation between protein levels of NFATC1 and DYRK1A in tissues was analyzed by Spearman's rank correlation test. *p* < 0.0001. rho = 0.0642. *n*=48. E. NFATC1 and DYRK1A were detected by WB in the cell lysate of NHA, U87, U251, T98G, and HEK293 cell lines. NFATC1 monoclonal antibody and DYRK1A polyclonal antibody were used as described in the methods section. F. Colocalization images for the immunofluorescent staining of DYRK1A and NFATC1 in U251 cells. DYRK1A was detected using a polyclonal anti‐DYRK1A antibody (CST, #2771), and NFATC1 was detected using a monoclonal antibody (Thermo Fisher, #MA3‐024), mouse IgG (Santa Cruz, sc‐2025), and rabbit IgG (Proteintech, B900610) as negative controls. Values represent means ±SD, *n* = 3.

Protein levels of DYRK1A and NFATC1 were examined in normal tissues and gliomas of different grades using tissue arrays and IHC analysis (Figure 1A, Table S1). IHC analysis showed higher expression of DYRK1A in gliomas, particularly in grade 4 gliomas (Figure 1B. *p < 0.001, glioma grade I vs. Normal; *p < 0.001, glioma grade II vs. Normal; *p < 0.001, glioma grade III vs. Normal; *p < 0.001, glioma grade IV vs. Normal;). Concomitantly, NFATC1 levels were also significantly higher in gliomas, especially in grade 4 gliomas than in normal tissues (Figure 1C. *p < 0.001, glioma grade I vs. Normal; *p < 0.001, glioma grade II vs. Normal; *p < 0.001, glioma grade III vs. Normal; *p < 0.001, glioma grade IV vs. Normal). DYRK1A was highly expressed in both the cytosol and nucleus of grade 4 glioma cells. High NFATC1 expression was also observed in grade 4 gliomas. NFATC1 expression was observed in both the cytosol and the nucleus. IHC analysis showed that most samples with positive DYRK1A staining also exhibited strong NFATC1 staining. Spearman's rank correlation test revealed a significant positive correlation between DYRK1A and NFATC1 protein levels (Figure 1D, *p* < 0.0001, Rho: 0.6042). DYRK1A and NFATC1 were highly expressed in a variety of glioma cell lines, including U87, U251, and T98G cells, compared to normal human astrocytes (NHA) and non‐glioma cell line HEK293 cells (Figure 1E, DYRK1A: 120.10% ± 0.86, *p < 0.01, U87 vs. NHA; 164.00% ± 0.90, *p < 0.001, U251 vs. NHA; 142.40% ± 1.61, *p < 0.001, T98G vs. NHA; 86.52% ± 1.02, *p < 0.01, HEK293 vs. NHA. NFATC1: 195.90% ± 1.81, *p < 0.001, U87 vs. NHA; 562.00% ± 1.71, *p < 0.001, U251 vs. NHA; 166.60% ± 3.23, *p < 0.001, T98G vs. NHA). NFATC1 expression was also correlated with DYRK1A expression in glioma cell lines. To further analyze the distribution of DYRK1A and NFATC1 in the cytoplasm and nucleus, we assessed their distribution in U251 cells using immunofluorescence staining. The results suggested that DYRK1A and NFATC1 were co‐localized in the cytoplasm and nucleus (Figure 1F).

### 3.2 Transcriptional expression of DYRK1A and NFATC1 in glioma, and their association with patient prognosis

**FIGURE 2:**
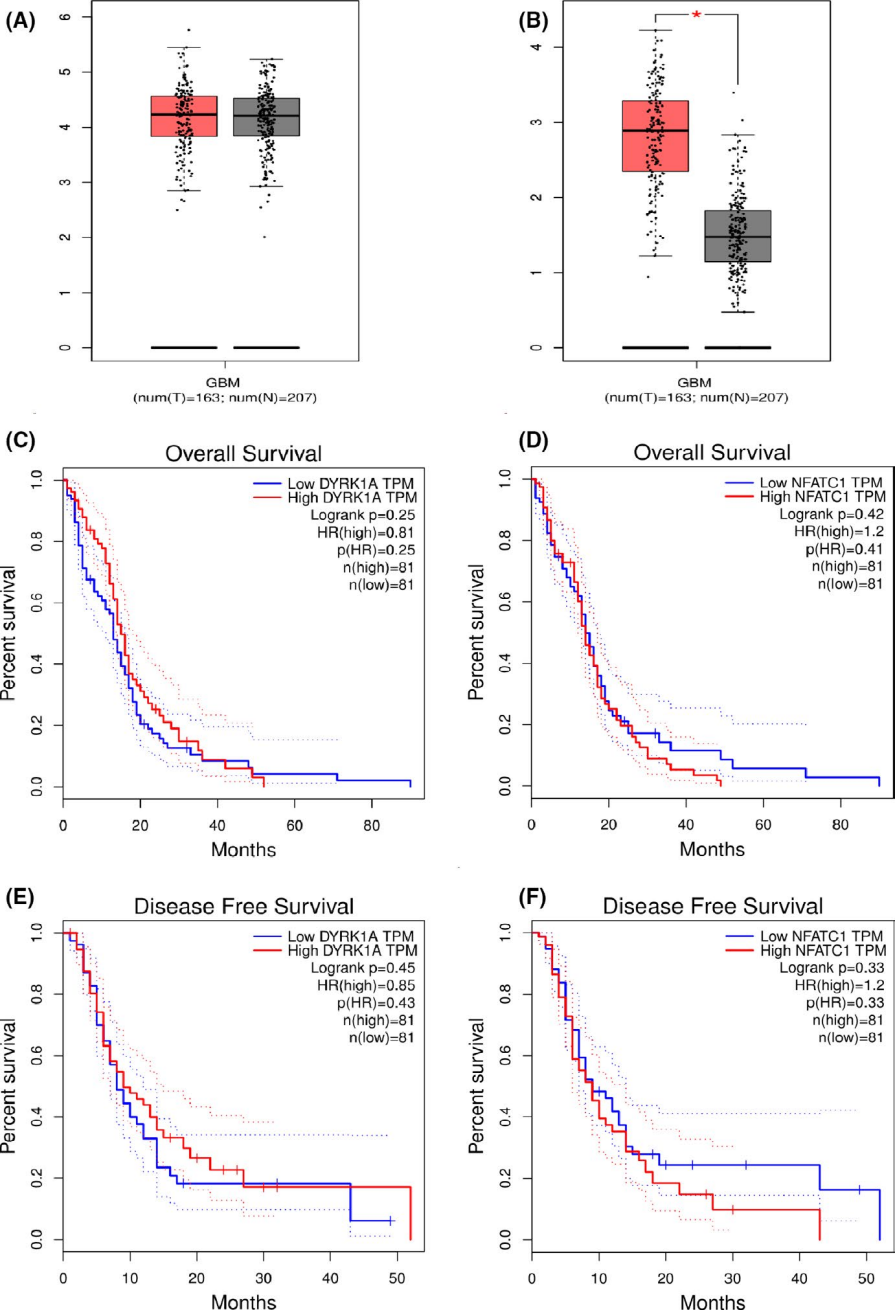
Transcriptional expression and prognostic significance of DYRK1A and NFATC1. A and B The expression of DYRK1A and NFATC1 was compared between T and N in TCGA GBM dataset. C–F. Kaplan–Meier plots were drawn for OS and DFS of patients stratified by tumoral expression of DYRK1A and NFATC1, respectively. P values were obtained by using the log‐rank test. Patients were stratified into low and high expression according to median values of DYRK1A and NFATC1 mRNA expression (<median vs. ≥median). GBM, glioblastoma multiforme; N, adjacent normal tissue; T, tumor tissue; TCGA, The Cancer Genome Atlas

By analyzing the expression profiles of DYRK1A and NFATC1 using RNA‐seq data from The Cancer Genome Atlas (TCGA), we found that there were no significant differences in DYRK1A transcription and an increase in NFATC1 transcription in GBMs (*n* = 163) compared to that in NTs (*n* = 207) (Figure 2A,B,^*^ *p* < 0.001).

To further confirm the adverse prognostic roles of DYRK1A and NFATC1 transcriptional expression in patients with GBM, we downloaded and analyzed DYRK1A or NFATC1 transcription data from TCGA and KM Plotter datasets. The results demonstrated that the transcriptional expression of DYRK1A and NFATC1 was not correlated with the overall survival (OS) and disease‐free survival (DFS) of the patients (Figure 2C–F).

### 3.3 DYRK1A increases NFATC1 protein expression and transcriptional activity

**FIGURE 3:**
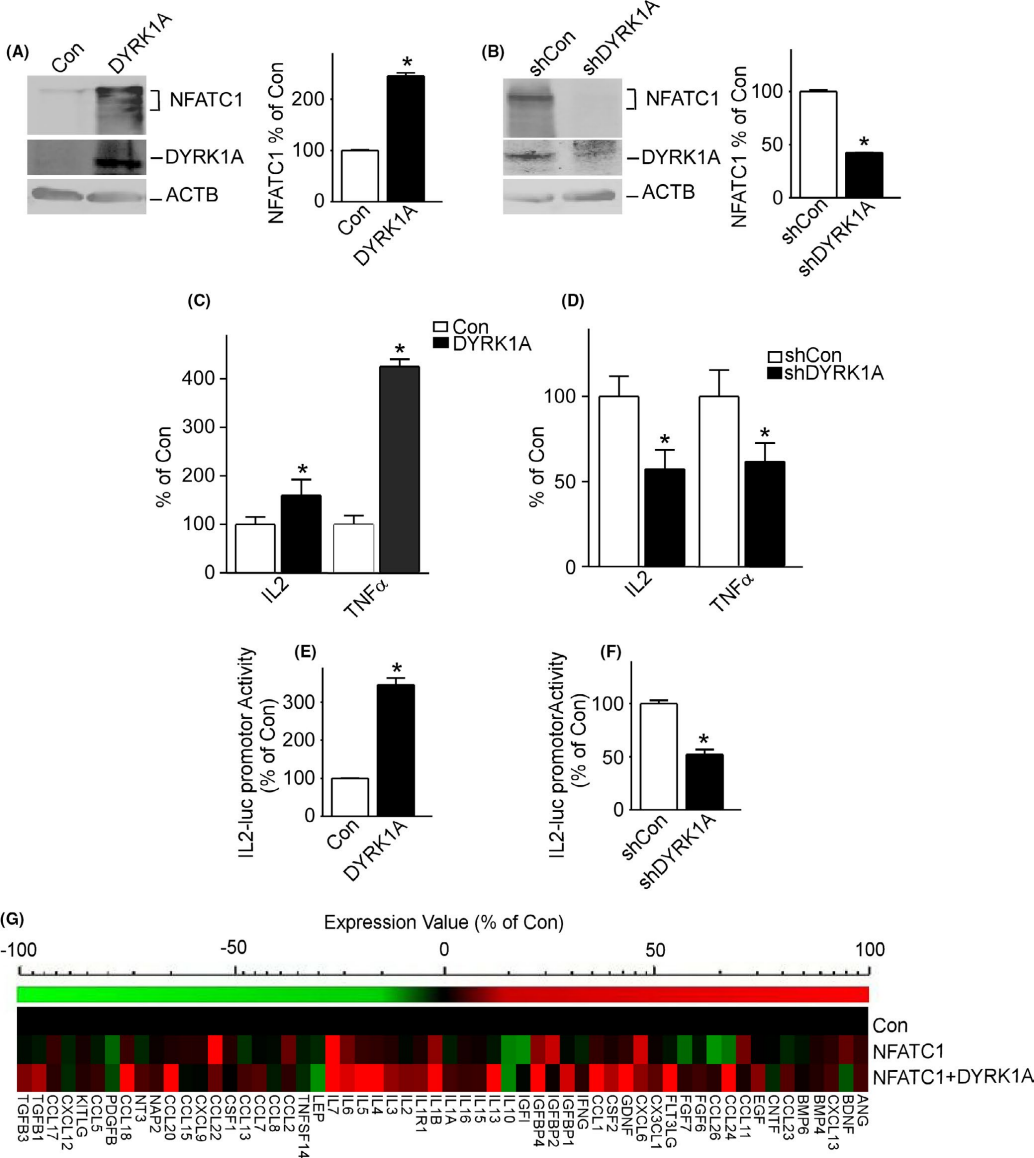
DYRK1A elevated NFATC1 protein expression and transcriptional activity. A, DYRK1A increased protein expression of NFATC1 in T98G cells. T98G cells were transfected with pCMV‐DYRK1A. NFATC1 and DYRK1A were detected by NFATC1 monoclonal antibody and DYRK1A polyclonal antibody. ACTB was used as the loading control. B, Inhibition of DYRK1A decreased protein expression of NFATC1 in T98G cells. T98G cells were transfected with pshDYRK1A. NFATC1 and DYRK1A were detected using NFATC1 monoclonal antibody and DYRK1A polyclonal antibody, respectively. ACTB was used as the loading control. C, NFATC1 target genes’ transcripts were increased by DYRK1A overexpression. T98G cells were cotransfected with p‐NFATC1mycflag and pCMV‐DYRK1A. qRT‐PCR was used to detect the mRNA expression of NFATC1 targets IL2 and TNFα. D, The mRNA levels of NFATC1 target genes IL2 and TNFα were decreased by DYRK1A knockdown. E, NFATC1 transcriptional activity was increased by DYRK1A. T98G cells were co‐transfected with NFATC1 activity reporter pNFATluc, p‐NFATC1mycflag, and pCMV‐DYRK1A. Dual luciferase assay was performed 48 h after transfection. F, Inhibition of DYRK1A decreased NFATC1 transcriptional activity. G, Cytokine array showed differential cytokine expression profiles regulated by DYRK1A in T98G cells. T98G cells were transfected with p‐NFATc1mycflag and pCMV‐DYRK1A. A RayBiotech human cytokine antibody array was performed to indicate transcriptional activity of NFATC1 48 h after transfection. Values represent means ±SD, *n* = 3. *p* < 0.05 by *student t*‐*test*

To examine whether NFATC1 is affected by DYRK1A in glioma cells, we measured the protein levels of NFATC1 in T98G cells. NFATC1 expression was increased to 245.7% ± 5.54 (*p < 0.001) by DYRK1A overexpression (Figure 3A), and decreased to 42.62% ± 0.04 (*p < 0.001) by DYRK1A knockdown (Figure 3). To investigate whether the altered protein levels of NFATC1 affected its transcriptional activity, the NFAT activity reporter plasmid, which contains the IL2 gene promoter region responsive to NFAT upstream of luciferase, was transfected into T98G cells, with Renilla luciferase pRL‐TK as a control. A dual luciferase assay indicated that overexpression of DYRK1A increased NFATC1 transcriptional activity to 345.6% ± 17.96 (*p < 0.001) (Figure 3E), while knockdown of DYRK1A decreased NFATC1 transcriptional activity to 52.2% ± 4.74 (*p < 0.01) (Figure 3F). To further confirm the effect of DYRK1A on NFATC1‐targeted genes, the expression levels of two NFATC1‐targeted genes, IL2 and TNFα, were measured in T98G cells by RT‐qPCR. The results showed that overexpression of DYRK1A greatly increased mRNA expression of IL2 and TNFα to 160.0% ± 24.11 (*p < 0.05) and 425.0% ± 14.81 (*p < 0.01), respectively (Figure 3C). Knockdown of DYRK1A decreased the mRNA expression of IL2 and TNFα to 57.3% ± 11.28 (*p < 0.05) and 61.8% ± 10.84 (*p < 0.05), respectively (Figure 3D). These results clearly demonstrate that DYRK1A increases NFATC1 transcriptional activity.

To further investigate whether the transcriptional activity of NFATC1 is affected by DYRK1A, cytokine arrays were performed on T98G cells transfected with NFATC1 and DYRK1A. Protein panels of various cytokines, including IGFBP4, IL1B, IL6, etc., were markedly increased by DYRK1A (Figure 3G, Table S2).

### 3.4 DYRK1A distinctively regulates NFATC1 and NFATC2

**FIGURE 4:**
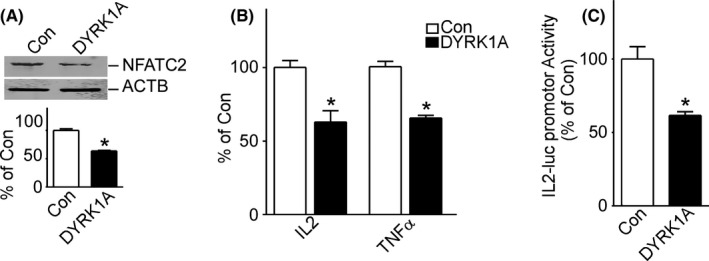
DYRK1A decreased NFATC2 protein expression and transcriptional activity. A, DYRK1A decreased protein expression of NFATC2 in T98G cells. T98G cells were transfected with pCMV‐DYRK1A. NFATC2 and DYRK1A were detected using NFATC2 monoclonal antibody and DYRK1A polyclonal antibody, respectively. ACTB was used as the loading control. Values represent means ±SD, *n* = 3. *p* < 0.05 by *student t*‐*test*. B, The mRNA levels of NFATC1 target genes IL2 and TNFα were decreased by DYRK1A overexpression. pWT‐NFATC2 was co‐transfected with pCMV‐DYRK1A into T98G cells. qRT‐PCR was used to detect NFATC2‐targeted gene expression of IL2 and TNFα. C, NFATC2 transcriptional activity was decreased by DYRK1A. T98G cells were co‐transfected with NFATC2 activity reporter pNFATluc, p‐NFATC2mycflag, and pCMV‐DYRK1A. Dual luciferase assay was performed 48 h after transfection. Values represent means ±SD, *n* = 3. *p* < 0.05 by *student t*‐*test*

Upregulation of NFATC1 by DYRK1A contradicts the effect of DYRK1A on NFATC2. To further verify our findings, we compared the effects of DYRK1A on NFATC1 and NFATC2. Consistent with previous reports, overexpression of DYRK1A markedly decreased NFATC2 protein levels to 63.79% ± 0.89 (*p < 0.001) (Figure 4A) and decreased the mRNA expression of IL2 and TNFα to 62.90% ± 7.7 (*p < 0.01) and 65.70% ± 1.80 (*p < 0.001), respectively (Figure 4B). Furthermore, overexpression of DYRK1A decreased NFATC2 transcriptional activity to 61.63% ± 2.45 (*p < 0.05) (Figure 4C). These results clearly indicate that DYRK1A decreases NFATC2 activity.

### 3.5 DYRK1A and NFATC1 synergistically increase glioma cell migration

**FIGURE 5:**
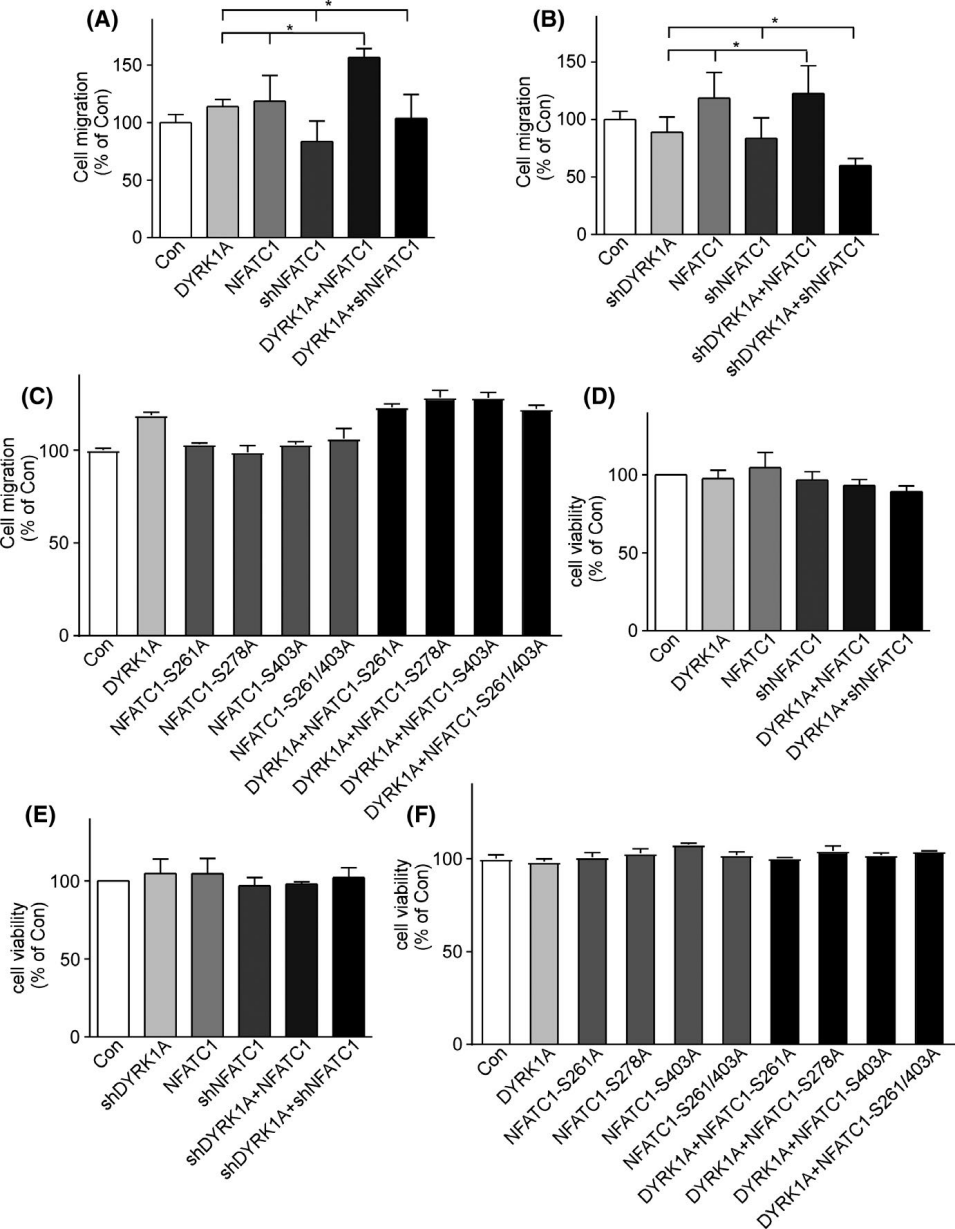
DYRK1A regulated NFATC1 to facilitate glioma cell migration. A, T98G cells were transfected with DYRK1A and NFATC1 expression vectors alone or simultaneously. Transwell assay showed that co‐expression of DYRK1A and NFATC1 greatly increased T98G cell migration. B, T98G cells were transfected with vectors knocking down DYRK1A and/or NFATC1. Simultaneous knockdown of DYRK1A and NFATC1 decreased T98G cell migration. C, T98G cells were transfected with DYRK1A or NFATC1 mutant expression vector alone or simultaneously. Transwell assay showed that co‐expression of DYRK1A and NFATC1 mutants marginally increased T98G cell migration. D, MTT assay showed that T98G cell viability was not affected by expression levels of DYRK1A and/or NFATC1. E, MTT assay showed that T98G cell viability was not affected by knockdown of DYRK1A and/or NFATC1. F, MTT assay showed that T98G cell viability was not affected by expression of DYRK1A and/or NFATC1 mutants. Values represent means ± SD, *n* = 3

To determine whether NFATC1 regulated by DYRK1A influences glioma cell migration, a transwell migration assay was performed on T98G cells. The results showed that DYRK1A or NFATC1 alone marginally increased T98G cell migration (columns 2 and 3 of Figure 5A), and co‐expression of DYRK1A and NFATC1 greatly increased T98G cell migration (156.70% ± 7.50 of control, *p < 0.001, column 4 of Figure 5A). In addition, although knockdown of DYRK1A or NFATC1 individually decreased T98G cell migration to 88.9% ± 13.26 and 83.7% ± 17.63, respectively (columns 2 and 3 of Figure 5B), knockdown of both reduced T98G cell migration to 59.92% ± 6.16 (*p < 0.01) (column 4 of Figure 5B). Knockdown of either DYRK1A or NFATC1 with overexpression of the other one did not affect T98G cell migration (column 6 of Figure 5A, column 6 of Figure 5B). However, NFATC1 mutants of the phosphorylation sites of DYRK1A alone did not affect T98G cell migration (columns 3, 4, 5, and 6 of Figure 5C), and co‐expression of DYRK1A and NFATC1 mutants marginally increased T98G cell migration as DYRK1A alone (columns 7, 8, 9, and 10 of Figure 5C).

To exclude the possibility that the effect on cell migration was due to cell proliferation, T98G cells were transfected with DYRK1A, NFATC1, their knockdown vectors, and NFATC1 mutants alone, or co‐transfected. The MTT assay showed that cell viability was not affected by DYRK1A and NFATC1 overexpression or knockdown (Figure 5D–F). Our data showed that DYRK1A and NFATC1 synergistically increased T98G cell migration.

### 3.6 NFATC1 peptides competitively inhibit DYRK1A and glioma cell migration

**FIGURE 6:**
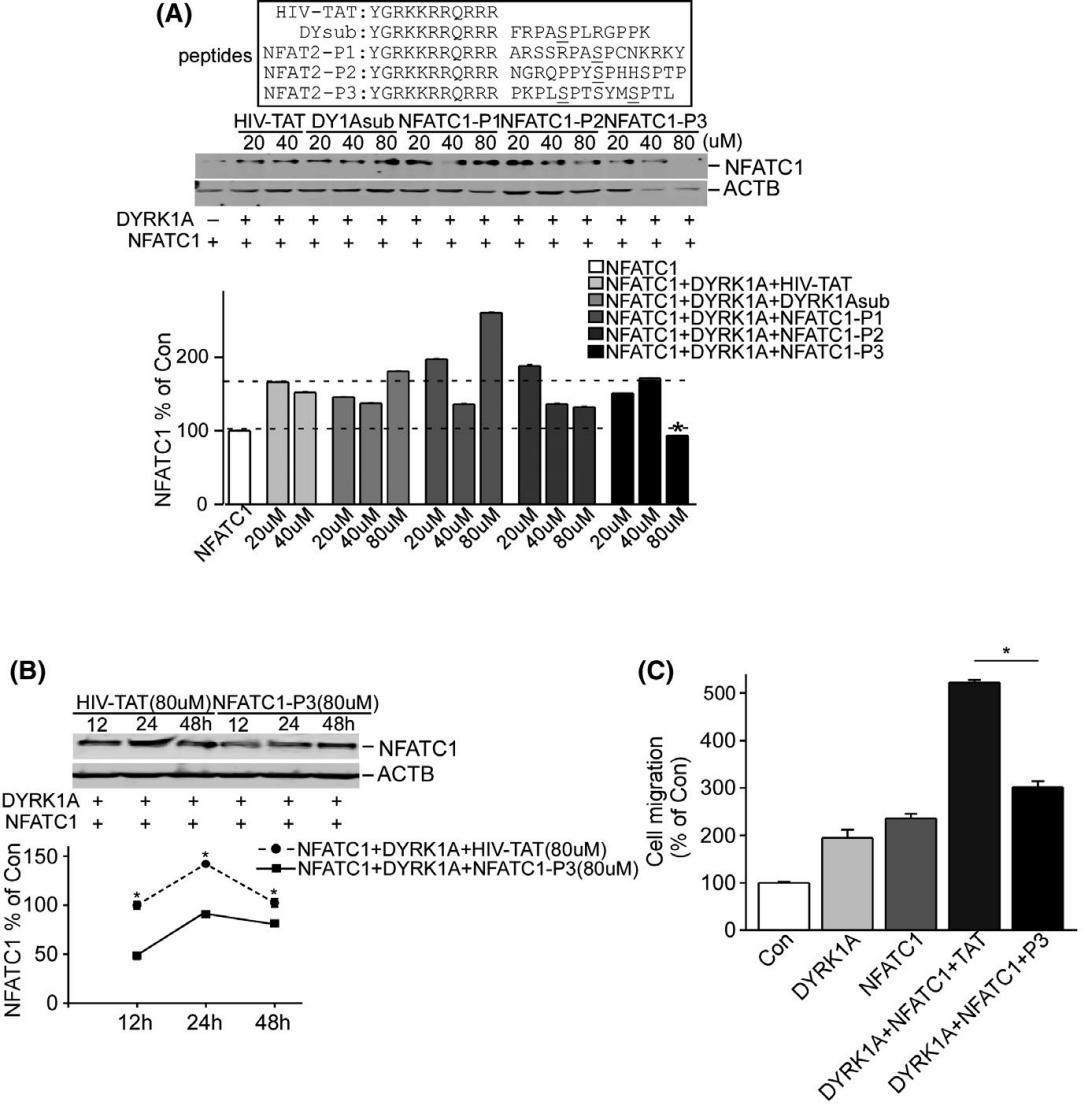
NFATC1‐P3 peptide decreased NFATC1 protein levels and inhibited T98G cell migration. A, T98G cells were transfected by p‐NFATC1mycflag and pCMV‐DYRK1A treated with different concentrations of peptides fused with an HIV‐TAT protein for 24 h after transfection, including HIV‐TAT, DYRK1A substrate, NFATC1‐P1, NFATC1‐P2, and NFATC1‐P3. Anti‐flag (M2) antibody was used to detect NFATC1 protein, and ACTB was used as loading control. B, NFATc1‐P3 inhibited increase in NFATC1 protein levels by DYRK1A at 12, 24, and 48 h. T98G cells were transfected by p‐NFATC1mycflag and pCMV‐DYRK1A and treated with HIV‐TAT (80 µM) and NFATC1‐P3 fusion polypeptides (80 µM) for 12, 24, and 48 h after transfection. NFATC1 protein was detected using anti‐flag (M2) antibody, and ACTB was used as the loading control. C, Transwell assay showed that NFATC1‐P3 peptide decreased T98G cell migration. T98G cells were cotransfected with p‐NFATC1mycflag and pCMV‐DYRK1A. After 24 h of transfection, cells were treated with HIV‐TAT (80 µM) and NFATC1‐P3 fusion polypeptides (80 µM). Values represent means ± SD, *n* = 3

The small molecule inhibitor harmine is an ATP‐competitive inhibitor of DYRK1A that lacks specificity and also inhibits other kinases.^32^ Nonetheless, the substrate inhibitor usually confers more specificity because the binding of kinase to its substrate relies on its conformation. To investigate whether the DYRK1A phosphorylation motifs of NFATC1 inhibit DYRK1A kinase activity, 15 amino acid peptides, including phosphorylation motifs 1, 2, and 3/4 (named NFATC1‐P1, NFATC1‐P2, NFATC1‐P3) were fused with an HIV‐TAT protein transduction domain capable of penetrating cell membranes. The results showed that NFATC1‐P3 significantly inhibited the increase in NFATC1 protein levels by DYRK1A at 80 µM concentration (Figure 6A. *p < 0.001). NFATC1‐P3 inhibited the increase in NFATC1 protein expression by DYRK1A at 12, 24, and 48 h (Figure 6B. *p < 0.001). To further investigate whether the NFATC1‐P3 peptide also affects glioma cell migration, NFATC1‐P3 was added to the cell media in the transwell migration assay. The results showed that NFATC1‐P3 peptides inhibited T98G cell migration from 522.4% ± 0.05 to 301.7% ± 0.12 (Figure 6C. *p < 0.001). These results indicate that the peptide competitively inhibits the effect of DYRK1A on NFATC1 and glioma cell migration.

## 4 DISCUSSION

Due to the high heterogeneity and biological properties of GBM cells, the treatment of GBM lacks progress. GBMs are traditionally considered a single histological entity. However, recent studies have revealed that the characterization of the genome, epigenome, and transcriptome of GBMs exhibit more frequent alterations and show a high degree of intratumoral and intratumoral heterogeneity.^33^, ^34^ The tumor microenvironment in which these tumor cells grow further increases this diversity.^35^, ^36^ The complex interaction between GBM cells and their surrounding microenvironment makes these tumors highly metastatic and invasive.^37^, ^38^ On the other hand, similar to other cancers, it has been proposed that tumor progression relies on cancer stem cells (CSCs), which are also responsible for tumor recurrence and resistance to treatment.^39^ Because of the complexity of GBMs, which are still poorly understood, we argued that it was necessary to re‐conceptualize glioblastoma therapy by studying the mechanism of tumor heterogeneity, CSC, and microenvironment in promoting tumor progression, invasion, and resistance to therapy.

Recent studies have revealed that NFAT is a transcription factor highly expressed in aggressive cancer cells and tissues, and mediates invasion through the transcriptional induction of pro‐invasion and pro‐migration genes. However, the mechanism of NFAT activation, especially the activation of NFATc1 to regulate the invasion of GBM cells, has not yet been fully elucidated. Our data demonstrate that NFATC1 and DYRK1A proteins are highly expressed in gliomas, particularly in grade 4 gliomas (GBM). Higher expression of NFATC1 and DYRK1A proteins was also observed in several glioma cell lines. Correlated expression of DYRK1A and NFATC1 proteins was observed in both glioma tissues and glioma cell lines. Moreover, DYRK1A and NFATC1 synergistically increased T98G glioma cell migration. Consistent with our results, Wang *et al*. showed that expression levels of NFATC1 are significantly higher in high‐grade gliomas than in low‐grade gliomas, and have the potential to regulate invasion in GBM.^40^ We previously showed that DYRK1A affects NFATC1 protein stability through phosphorylation of NFATC1 at S261, S278, S403, and S409. Our data suggest a synergistic effect of DYRK1A and NFATC1 on glioma cell migration and tumor metastasis. From the analysis of TCGA and KM Plotter datasets, we found that the transcriptional expression of DYRK1A and NFATC1 was not associated with OS and DFS in patients, even though the transcriptional expression of NFATC1 was elevated in GBMs. However, both DYRK1A and NFATC1 were highly expressed in tissues and cells of GBM, and the protein expression levels of DYRK1A and NFATC1 were positively correlated. Due to the limitation of samples, we could not analyze the correlation of DYRK1A and NFATC1 protein expression levels with the OS and DFS of GBM patients. In summary, the roles of DYRK1A and NFATC1 in GBM are better reflected at the protein level, which is consistent with our previous study showing that DYRK1A acts as a kinase at the protein level to regulate the target protein NFATC1.^30^, ^41^ The increased transcriptional expression of the NFATC1 gene in GBMs may be due to the auto‐regulation of increased NFATC1 protein caused by DYRK1A kinase in GBMs.^42^, ^43^

NFATs were originally discovered as transcription factors involved in the development and activation of lymphocytes and differentiation of cardiomyocytes.^44^, ^45^, ^46^ However, in recent years, accumulating evidence has shown the role of NFATs in carcinogenesis. Although NFAT family proteins, apart from NFAT5, exhibit high sequence similarity and conservatism, they are not redundant in function. In particular, they have different effects on tumorigenesis and cancer progression. In the activation process of T‐cells and B‐cells, NFATC1 is continuously active, protecting lymphocytes from activation‐induced cell death, whereas NFATC2 inhibits lymphocyte proliferation and promotes lymphocyte activation‐induced cell death.^47^ Activated NFATC2 in fibroblast NIH3T3 cells may block the cell cycle and inhibit cell transformation, showing potential to suppress the tumor, while activated NFATC1 promotes cell proliferation and malignant transformation of carcinogenic properties.^48^ Activation of NFATC1 has been demonstrated in tumor tissues of patients with diffuse large B‐cell lymphoma (DLBCL) and cell lines originating from lymphoma.^49^, ^50^, ^51^ Studies on the mechanism of NFATC1 in GBM have reported that NFATc1 promotes U251 cell invasion through the induction of PTGS2.^40^ In summary, NFATC2 acts as a tumor suppressor, whereas NFATC1 exhibits oncogenic activity.^19^, ^20^ Our results showed distinct effects of DYRK1A on NFATC1 and NFATC2. DYRK1A increased the protein levels and transcriptional activity of NFATC1 and decreased those of NFATC2. NFATC1 and its family members have a very complex regulation and regulated mode in tumors, including GBM. Different kinases have multiple regulatory methods for the same member of the family, and differently activated members have different functions. Our results provide some support for the function of NFATC1 in GBM, and further studies are needed to determine the expression levels of all NFAT family members in glioma as well as their functions and mechanisms in tumorigenesis and tumor migration.

DYRK1A inhibitors have been described previously, such as harmine, INDY, KH‐CB19, leucettines, and pyridinylthiophene.^52^, ^53^, ^54^, ^55^, ^56^ However, they have limited *in vivo* use due to their lack of specificity, as they also target other enzymes, including monoamine oxidase. From the phosphorylation motifs on NFATC1, we synthesized several peptide inhibitors of DYRK1A. Our results show that inhibition of DYRK1A by NFATC1‐P3 polypeptides significantly destabilizes NFATC1 protein levels and reduces T98G glioma cell migration. Our studies imply that more specific DYRK1A inhibitors may be developed based on molecular mechanisms. Further studies are needed to test the potential anti‐cancer effects of these peptide inhibitors *in vivo*.

In conclusion, DYRK1A activated NFATC1 and increased glioblastoma migration. Polypeptide pharmacological inhibition of DYRK1A may represent a promising therapeutic intervention for GBM.

## DATA AND MATERIALS AVAILABILITY STATEMENT

The raw materials and data are available.

## CONFLICT OF INTERESTS

The authors declare no potential conflicts of interest.

## AUTHORS’ CONTRIBUTIONS

XS and HL conceived and designed the experiments; HL and QS performed the experiments; SC, LC, WJ, and JZ analyzed and contributed reagents/materials/analysis tools; XS and HL wrote the paper.

## ETHICS APPROVAL AND CONSENT TO PARTICIPATE

All animal protocols were approved by the Institutional Ethics Committee on Animal Research of Qilu Hospital of Shandong University.

## CONSENT FOR PUBLICATION

All authors reviewed the manuscript and consent to publish in this journal.

## Supporting information

Table S1‐S2
