# Supplementary material for: DYRK1A activates NFATC1 to increase glioblastoma migration
Source: Cancer Med. 2021 Jul 26;10(18):6416–27. doi: 10.1002/cam4.4159 (PMC8446559; doi:10.1002/cam4.4159)
Supplement: Supplementary file 1 — Table S1‐S2 [file CAM4-10-6416-s001.docx]

**Supplements**

**Table1. Specification sheet for brain cancer and normal tissue microarray.** 44 cases of brain tumor (diffuse astrocytoma, anaplastic astrocytoma and glioblastoma) and 10 normal tissues were used in the IHC study.

| **No.** | **Sex** | **Age** | **Organ** | **Pathology diagnosis** | **Grade** | **Type** |
| --- | --- | --- | --- | --- | --- | --- |
| 1 | M | 47 | Brain | Diffuse astrocytoma | 1 | Malignant |
| 2 | F | 31 | Brain | Diffuse astrocytoma | 1 | Malignant |
| 3 | F | 56 | Brain | Diffuse astrocytoma | 1 | Malignant |
| 4 | M | 21 | Brain | Diffuse astrocytoma | 1 | Malignant |
| 5 | M | 12 | Brain | Diffuse astrocytoma | 1-2 | Malignant |
| 6 | F | 41 | Brain | Diffuse astrocytoma | 1 | Malignant |
| 7 | F | 32 | Brain | Diffuse astrocytoma | 1 | Malignant |
| 8 | M | 42 | Brain | Diffuse astrocytoma | 1 | Malignant |
| 9 | F | 51 | Brain | Diffuse astrocytoma | 2 | Malignant |
| 10 | M | 50 | Brain | Diffuse astrocytoma | 2 | Malignant |
| 11 | M | 37 | Brain | Diffuse astrocytoma | 2 | Malignant |
| 12 | M | 51 | Brain | Diffuse astrocytoma | 2 | Malignant |
| 13 | F | 42 | Brain | Diffuse astrocytoma | 2 | Malignant |
| 14 | F | 27 | Brain | Diffuse astrocytoma | 2 | Malignant |
| 15 | M | 56 | Brain | Diffuse astrocytoma | 2 | Malignant |
| 16 | M | 56 | Brain | Diffuse astrocytoma | 2 | Malignant |
| 17 | M | 56 | Brain | Astrocytoma (tumoral necrosis) | – | Malignant |
| 18 | F | 45 | Brain | Diffuse astrocytoma | 2 | Malignant |
| 19 | M | 53 | Brain | Diffuse astrocytoma | 1 | Malignant |
| 20 | M | 50 | Brain | Diffuse astrocytoma | 2 | Malignant |
| 21 | M | 66 | Brain | Diffuse astrocytoma | 2 | Malignant |
| 22 | M | 46 | Brain | Diffuse astrocytoma | 2 | Malignant |
| 23 | F | 49 | Brain | Diffuse astrocytoma | 2 | Malignant |
| 24 | M | 49 | Brain | Diffuse astrocytoma | 2 | Malignant |
| 25 | M | 52 | Brain | Anaplastic astrocytoma | 2-3 | Malignant |
| 26 | F | 40 | Brain | Anaplastic astrocytoma | 3 | Malignant |
| 27 | M | 10 | Brain | Anaplastic astrocytoma | 3 | Malignant |
| 28 | F | 35 | Brain | Anaplastic astrocytoma | 3 | Malignant |
| 29 | M | 59 | Brain | Glioma sarcomatosum | – | Malignant |
| 30 | F | 47 | Brain | Anaplastic astrocytoma | 3 | Malignant |
| 31 | M | 42 | Brain | Anaplastic astrocytoma | 3 | Malignant |
| 32 | M | 59 | Brain | Anaplastic astrocytoma | 3 | Malignant |
| 33 | M | 76 | Brain | Diffuse astrocytoma | 2 | Malignant |
| 34 | F | 40 | Brain | Glioblastoma | 4 | Malignant |
| 35 | M | 23 | Brain | Glioblastoma | 4 | Malignant |
| 36 | M | 64 | Brain | Glioblastoma | 4 | Malignant |
| 37 | F | 22 | Brain | Glioblastoma | 4 | Malignant |
| 38 | M | 43 | Brain | Glioblastoma | 4 | Malignant |
| 39 | M | 39 | Brain | Glioblastoma | 4 | Malignant |
| 40 | M | 9 | Brain | Glioblastoma | 4 | Malignant |
| 41 | M | 49 | Cerebrum | Astrocytoma of ventricle | 2 | Malignant |
| 42 | F | 58 | Cerebrum | Astrocytoma of right frontal lobe | 3 | Malignant |
| 43 | M | 38 | Cerebrum | Glioblastoma of right tempral lobe | 4 | Malignant |
| 44 | M | 43 | Cerebrum | Anaplastic oligodendroglioma of left frontal lobe | 3 | Malignant |
| 45 | F | 15 | Cerebrum | Normal Cerebrum tissue | – | Normal |
| 46 | F | 21 | Cerebrum | Normal Cerebrum tissue | – | Normal |
| 47 | F | 21 | Brain | Normal brain tissue | – | Normal |
| 48 | F | 21 | Brain | Normal brain tissue | – | Normal |
| 49 | F | 38 | Brain | Normal brain tissue | – | Normal |
| 50 | F | 42 | Brain | Normal brain tissue | – | Normal |
| 51 | F | 2 | Brain | Normal brain tissue (hyperplasia of gliocyte) | – | Normal |
| 52 | M | 26 | Brain | Normal brain tissue | – | Normal |
| 53 | M | 38 | Brain | Normal brain tissue | – | Normal |
| 54 | M | 45 | Brain | Normal brain tissue | – | Normal |

**Table2. The cytokine array showed differential cytokine expression profile regulated by DYRK1A in T98G cells.** T98G cells were transfected with pWT-NFAT1 or pWT-NFAT1 and pCMV-DYRK1A. RayBiotech human cytokine antibody array was performed to indicate transcriptional activity of NFATs 48 hours after transfection. Values represent means±SD, n=3. Formula= (Sample/ Negative-1)*100%.

| Gene name | NFATc1 | NFATc1+DYRK1A |
| --- | --- | --- |
| ANG | 8.255946 | 10.87439 |
| BDNF | 15.86785 | -13.6881 |
| CXCL13 | 6.887852 | 10.98775 |
| BMP4 | 4.489001 | 5.53692 |
| BMP6 | -3.04536 | 2.118551 |
| CCL23 | -5.9868 | 13.56343 |
| CNTF | -0.40334 | -6.80108 |
| EGF | 0.36456 | 21.63584 |
| CCL11 | 18.49206 | 4.186712 |
| CCL24 | -18.8782 | 53.27664 |
| CCL26 | -24.069 | 6.470043 |
| FGF6 | -3.00095 | 10.15459 |
| FGF7 | -16.8815 | 7.178086 |
| FLT3LG | -3.13806 | 108.8743 |
| CX3CL1 | 1.860923 | 14.90119 |
| CXCL6 | 34.05971 | 23.18148 |
| GDNF | 7.559708 | 64.00086 |
| CSF2 | 4.114038 | 28.62127 |
| CCL1 | 8.135999 | 80.66998 |
| IFNG | -5.08884 | 6.818965 |
| IGFBP1 | -1.1414 | 34.19347 |
| IGFBP2 | 32.57253 | 2.121428 |
| IGFBP4 | 22.38471 | 105.3794 |
| IGFI | -22.605 | -0.84856 |
| IL10 | -21.2117 | -22.0826 |
| IL13 | -3.46514 | 56.41184 |
| IL15 | 2.388634 | 9.547459 |
| IL16 | 3.503447 | 9.029759 |
| IL1A | -4.43622 | 11.21845 |
| IL1bB | 23.13671 | 40.51148 |
| IL1R1 | 4.757613 | 20.94214 |
| IL2 | -1.07614 | 20.50213 |
| IL3 | 5.612359 | 24.14583 |
| IL4 | 7.513351 | 45.99518 |
| IL5 | 8.625421 | 54.3052 |
| IL6 | 18.32373 | 35.8203 |
| IL7 | 53.63996 | 50.25772 |
| LEP | -6.37919 | -23.2178 |
| TNFSF14 | -3.89378 | -6.86569 |
| CCL2 | 16.65266 | 7.168312 |
| CCL8 | -1.43628 | -4.14958 |
| CCL7 | -0.6971 | 13.32341 |
| CCL13 | -7.63956 | 10.92822 |
| CSF1 | 6.049201 | 0.361431 |
| CCL22 | 47.42432 | 16.06982 |
| CXCL9 | 5.47353 | -0.76325 |
| CCL15 | 5.690925 | -1.36967 |
| CCL20 | 4.657157 | 46.12344 |
| NAP2 | 1.934376 | 9.041446 |
| NT3 | -6.25224 | 12.46942 |
| CCL18 | 9.276492 | 47.02034 |
| PDGFB | -16.2538 | -11.6747 |
| CCL5 | -3.97911 | 8.552965 |
| KITLG | 3.762553 | 5.745913 |
| CXCL12 | -5.5108 | -6.54365 |
| CCL17 | 8.816906 | 6.739276 |
| TGFB1 | -3.65946 | 27.3658 |
| TGFB3 | -1.71301 | 20.4604 |
